# Chikungunya virus time course infection of human macrophages reveals intracellular signaling pathways relevant to repurposed therapeutics

**DOI:** 10.7717/peerj.13090

**Published:** 2022-03-21

**Authors:** Madison Gray, Israel Guerrero-Arguero, Antonio Solis-Leal, Richard A. Robison, Bradford K. Berges, Brett E. Pickett

**Affiliations:** 1 Microbiology and Molecular Biology, Brigham Young University, Provo, Utah, United States of America; 2 Population Health and Host-pathogen Interactions Programs, Texas Biomedical Research Institute, San Antonio, Texas, United States of America

**Keywords:** Chikungunya virus, Transcriptomics, Signaling pathways, Drug repurposing, Macrophage, Virology

## Abstract

**Background:**

*Chikungunya virus* (CHIKV) is a mosquito-borne pathogen, within the *Alphavirus* genus of the *Togaviridae* family, that causes ~1.1 million human infections annually. CHIKV uses *Aedes albopictus* and *Aedes aegypti* mosquitoes as insect vectors. Human infections can develop arthralgia and myalgia, which results in debilitating pain for weeks, months, and even years after acute infection. No therapeutic treatments or vaccines currently exist for many alphaviruses, including CHIKV. Targeting the phagocytosis of CHIKV by macrophages after mosquito transmission plays an important role in early productive viral infection in humans, and could reduce viral replication and/or symptoms.

**Methods:**

To better characterize the transcriptional response of macrophages during early infection, we generated RNA-sequencing data from a CHIKV-infected human macrophage cell line at eight or 24 hours post-infection (hpi), together with mock-infected controls. We then calculated differential gene expression, enriched functional annotations, modulated intracellular signaling pathways, and predicted therapeutic drugs from these sequencing data.

**Results:**

We observed 234 pathways were significantly affected 24 hpi, resulting in six potential pharmaceutical treatments to modulate the affected pathways. A subset of significant pathways at 24 hpi includes AGE-RAGE, Fc epsilon RI, Chronic myeloid leukemia, Fc gamma R-mediated phagocytosis, and Ras signaling. We found that the MAPK1 and MAPK3 proteins are shared among this subset of pathways and that Telmisartan and Dasatinib are strong candidates for repurposed small molecule therapeutics that target human processes. The results of our analysis can be further characterized in the wet lab to contribute to the development of host-based prophylactics and therapeutics.

## Introduction

Chikungunya virus (CHIKV) infects approximately 1.1 million people per year in over 100 countries worldwide (Goupil & Mores, 2016), with over one billion people at-risk of becoming infected with the virus due to the presence of the required mosquito vector in tropical and subtropical climates (Nasci, 2014; Nsoesie et al., 2016; Goupil & Mores, 2016). CHIKV infections were primarily observed in India prior to 2015 when it emerged in the Western Hemisphere, which may be at least partially attributable to the role of *Aedes albopictus* mosquitoes as active transmission vectors in addition to *Aedes aegypti*, which had been considered the primary vector (Pagès et al., 2009; Delatte et al., 2010). CHIKV is an arthrogenic alphavirus, which can cause chronic rheumatoid arthritis-like symptoms in the peripheral joints of some patients, likely caused by viral presence in the joint tissue (Nakaya et al., 2012; Lounibos & Kramer, 2016). The name of the virus itself means “that which bends up”, referring to the painful posture of those who develop chronic sequelae after acute infection. Currently, no cure, treatment, or vaccine exists for most alphaviruses, including CHIKV.

The host immune response, which includes the rapid recruitment of macrophages, is initiated shortly after the virus infects a human *via* a mosquito vector (Haist et al., 2017). CHIKV initially invades stromal cells bypassing their intracellular defense mechanisms to induce apoptosis (Srivastava et al., 2020). Cellular apoptosis is advantageous to the virus which is ensconced within the produced apoptotic blebs that get consumed by phagocytic cells including macrophages (Srivastava et al., 2020). Within the monocytes, the virus is stabilized and begins to replicate preceding the acute phase of infection (Sourisseau et al., 2007; Wikan et al., 2012; Srivastava et al., 2020). Infected macrophages then operate as transporters for the virus, collecting in peripheral tissues, and serving as a possible mechanism for the severe joint pain associated with CHIKV infection (Her et al., 2010; Srivastava et al., 2020). Chronic CHIKV-infections still have detectable amounts of infected macrophage cells in their synovial tissue (Her et al., 2010). This process enables the virus to go through multiple rounds of infection in macrophages and other cells, which contributes to the onset of symptoms and pathogenesis. As such, maximizing the antiviral response in the macrophage to reduce virion production and/or escape during acute infection could lead to a host-based therapeutic strategy with smaller risks of the emergence of resistance in the future—especially when compared to antiviral strategies that strictly target viral proteins.

The aims of the current study are two-fold: (1) to gain a better understanding of the intracellular transcriptional response to CHIKV infection in human macrophages at 8 and 24 hpi, and (2) to apply that mechanistic knowledge to predict existing drugs that could be repurposed as either prophylactic or therapeutic treatments to minimize viral replication and spread from macrophages. Specifically, we calculated statistically significant differentially expressed genes (DEGs), functional gene annotations, and intracellular signaling pathways to improve our understanding of how macrophages respond to CHIKV infection. We then mined this information to predict existing therapeutic treatments that could be repurposed to reverse key host-pathogen interactions to reduce virus replication, infection, and pathogenesis. To our knowledge, this is the first RNA-seq experiment involving CHIKV infection of cultured human macrophages. The results obtained from this study contribute to ongoing efforts to develop effective prophylactics and/or therapeutic treatments.

## Materials and Methods

### Cell infection

Macrophages were differentiated from the U937* human monocyte cell line and infected with CHIKV as described previously (Guerrero-Arguero et al., 2020). Briefly, monocytes were propagated with fetal bovine serum (FBS), Penicillin/Streptomycin, L-glutamine, and HEPES buffer. The cells were then cultured in T-75 culture flasks at 37 °C before being transferred to six-well tissue culture plates. Cells were then induced to become adherent macrophages by exposing them to phorbol 12-mystrate 13-acetate (PMA) and incubating in 3 mL of RPMI 1640 complete media at 37 °C for 24 h. PMA-differentiated U937 monocyte-derived macrophages were then transferred to 12-well tissue culture plates prior to infection with the CHIKV-LR strain at 0.1 multiplicity of infection (MOI) and incubated at 37 °C with 5% CO_2_ for 2 h. The virus-containing media was then removed prior to washing the cells three times with PBS and adding fresh media prior to incubation. Mock-infected cells were incubated using the same protocol and reagents, and only lacked the presence of virus.

### RNA extraction and RNA-sequencing

Biological replicates, in duplicate, of CHIKV-infected and mock-infected macrophages were harvested at eight and 24 h post-infection (hpi) by removing the supernatant and washing with PBS. RNA was extracted from all duplicate samples using Trizol reagent according to the manufacturer’s protocol as performed previously (Guerrero-Arguero et al., 2020). A NanoDrop instrument was then used to quantify the concentration of RNA as approximately 1.8 ng/µL for each sample. Reverse transcription of the RNA into cDNA was then performed prior to generating Nextera XT sequencing libraries and samples were barcoded for multiplexing. The concentration of post-capture libraries for the sample pools ranged from 0.02 to 0.3 ng/µL. An Illumina NovaSeq instrument was then used to produce paired-end 150 bp reads for each sample for downstream analysis. An average of ~40 million reads were collected from the control samples, with ~11 million collected from the eight hpi samples and ~188 million collected from the 24 hpi samples.

### Statistical power analysis

The Scotty tool was used to confirm the sequencing depth and number of samples were above what was necessary to achieve sufficient statistical power (Busby et al., 2013).

### RNA-seq data preprocessing

The fastq files containing the RNA-sequencing data were subjected to analysis by the Snakemake-based Automated Reproducible MOdular Workflow for Preprocessing and Differential Analysis of RNA-seq Data (ARMOR) software. Specifically, the fastq files, associated metadata, and a configuration file for each dataset were used as input to the ARMOR workflow (Orjuela et al., 2019). This workflow, which uses the python-based Snakemake workflow language (Köster & Rahmann, 2018), performs the following steps: trimming reads with TrimGalore (Krueger et al., 2021), performing quality control with FastQC (s-andrews, 2021), mapping and quantifying reads to the human GRCh38 transcriptome with Salmon (Patro et al., 2017), generating DEG lists with edgeR (Robinson, McCarthy & Smyth, 2010), performing GO enrichment with Camera (Wu & Smyth, 2012), and identifying significant splice variants from the detected transcripts with DRIMseq (Nowicka & Robinson, 2016).

### Signaling pathway enrichment

The DEGs from ARMOR were then used as input to the signaling pathway impact analysis (SPIA) algorithm to identify intracellular signaling pathways that were significantly enriched with DEGs (Tarca et al., 2009). SPIA implements a bootstrapping approach to generate a null distribution for DEGs in each pathway, then uses the null distribution to calculate the significance threshold. Over 1,500 public signaling pathways from the KEGG, Reactome, NCI, BioCarta, and Panther databases were used to test for enrichment. The regulatory patterns from these analyses were then cross-referenced to the Open Targets (opentargets.org) database to identify known drug targets that were present in each of the significant pathways, and which could be repurposed as host-based therapeutics for CHIKV.

### Drug repurposing and ranking

To increase the number of high-confidence potential therapeutics, a ranking strategy was implemented. For this study, a focus on small molecules was justified to facilitate ease of therapeutic distribution in geographical regions lacking adequate cold-storage resources. To simplify the multiple characteristics that were considered in the ranking of the results, a numerical score that represents a variety of metrics was assigned to each result. Contributing factors to this score include but are not exclusive to: whether the drug has been through clinical trials or has obtained US Food and Drug Administration (FDA) approval, if the drug had been approved and tested in multiple studies, the toxicity of the drug (LD_50_ values and/or recommended diet restrictions, manufacturer safety statements, requirements of administration, and long-term impacts or risks that are included in drug consumption), prior publications or studies discussing the drug’s effectiveness against CHIKV, and whether any complications were indicated during trials—either during or after FDA approval.

## Results

### Power analysis

We began by confirming that our experimental design included appropriate numbers of reads and biological replicates to achieve sufficient statistical power. Using two biological replicates and at least 10 million reads, our analysis showed that ~35% of genes could be detected at a log_2_ fold-change of ≥1.6 with a *p*-value of <0.05. We observed the replicate dispersion among mock-infected samples was 0.89, while that for the 24 hpi samples was 0.44. In addition, hierarchical clustering grouped the mock-infected samples together and the 24 hpi samples together. We calculated that ~55% of genes could be detected with a log_2_ fold-change of >3, which is acceptable. We calculated the measurement bias, the percentage of genes measured with at least 95% of the maximum power to be ~94%, which is also within the acceptable range.

### Differential expression and gene ontology enrichment shows lag in immune response

**Figure 1:**
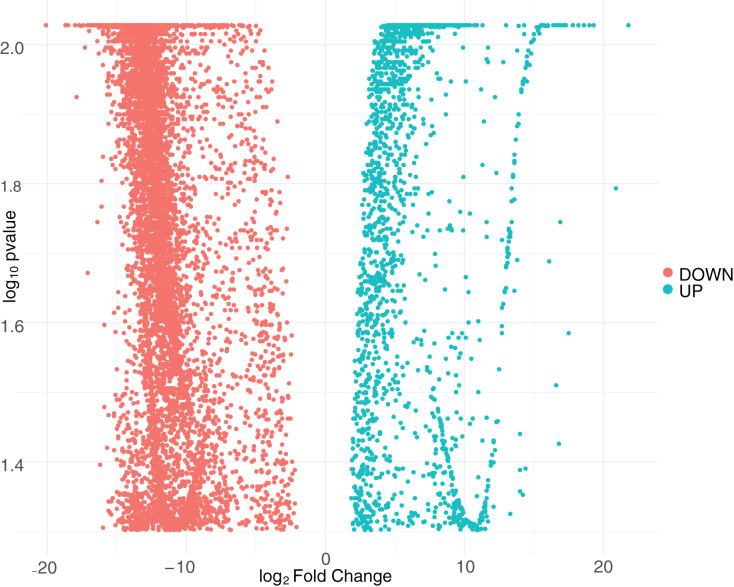
Volcano plot of differentially expressed genes at 24 hpi with CHIKV. The distribution of adjusted *p*-values and fold-change values for up-regulated genes (green) or down-regulated genes (red) at 24 hpi with CHIKV is displayed.

We first quantified the intracellular transcriptional response of human macrophages to CHIKV infection by calculating the log2 fold-change and false discovery rate (FDR) adjusted *p*-values for DEGs. We constructed these comparisons to compare the samples from CHIKV-infected macrophages at 8 and 24 hpi to the mock-infected cells. We plotted the relationship between all detected genes and retained those with a FDR *p*-value smaller than 0.05. Interestingly, we found no genes that surpassed our significance threshold of 0.05 for the eight hpi comparison, so this comparison was ignored in subsequent analyses. This may be at least partially due to the lower number of reads generated for the eight hpi samples. In contrast, we observed 9,670 genes that had significant differential expression at 24 hpi (Supplemental File 1), with a majority of DEGs being downregulated during early infection (Fig. 1). A subset of the most significant genes that were impacted by virus infection at 24 h post-infection included those that code for extracellular mucins (MUC3A, MUC12, MUC5B), an immunoglobulin gene (IGFN1), a nucleosome assembly protein (NAP1L1), and Ras protein activator (RASA4) as a Calcium signaling component.

We performed a Gene Ontology (GO) enrichment analysis on the DEG list to identify the cellular component, molecular mechanism, and/or biological process annotations that are assigned to each DEG result from the CHIKV-infected macrophages. The 24 hpi GO analysis identified eight statistically significant GO terms (Supplemental File 2). Significant GO terms include metal ion binding, nucleic acid binding, cell adhesion and extracellular matrix organization.

### Signaling pathway enrichment reveals intracellular stress

**Figure 2:**
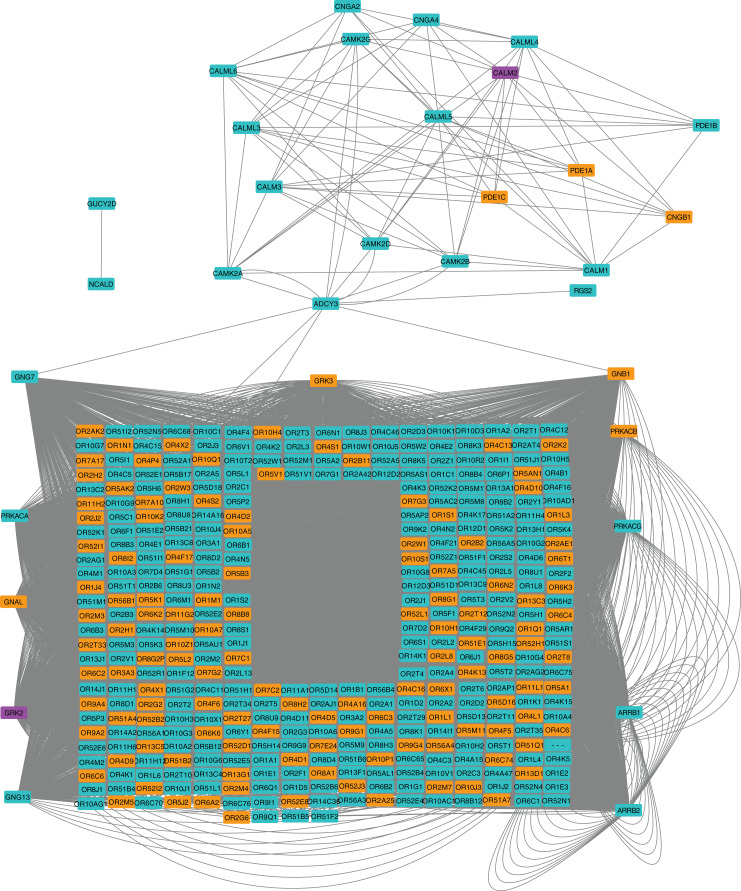
Protein-protein interaction network for the Olfactory Transduction pathway at 24 hpi with CHIKV. Nodes in orange are members of the pathway that are significantly downregulated. Nodes in purple are members of the pathway that are significantly upregulated. Nodes in cyan are unaffected members of the pathway.

**Figure 3:**
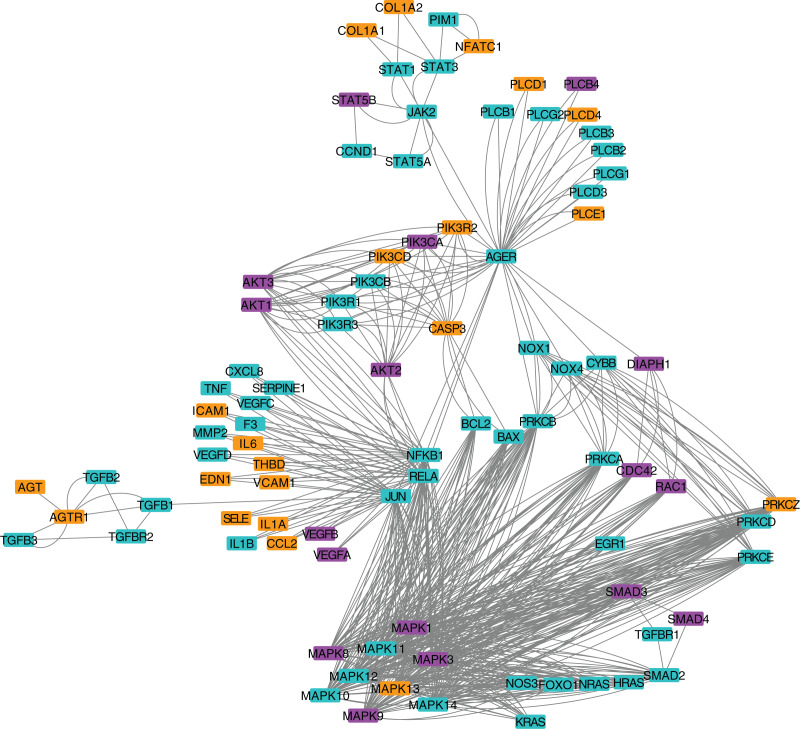
Protein-protein interaction network for the AGE-RAGE pathway. Nodes in orange are members of the pathway that are significantly downregulated. Nodes in purple are members of the pathway that are significantly upregulated. Nodes in cyan are unaffected members of the pathway.

**Figure 4:**
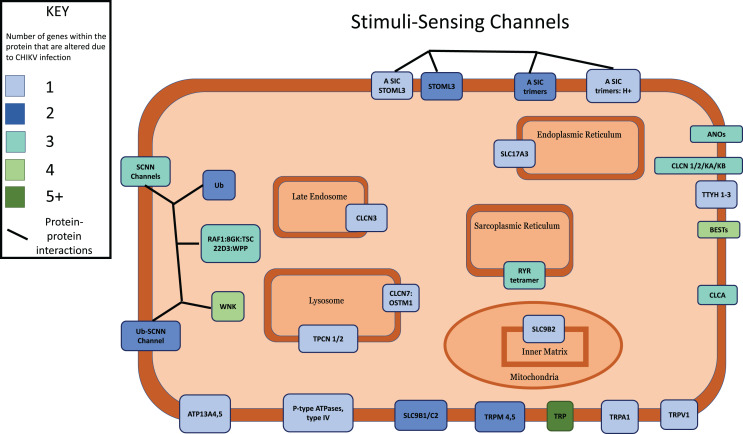
Multi-protein complexes contribute to the Stimuli-Sensing Channels intracellular signaling pathway. Differentially expressed genes with a *p*-value < 0.05 were mapped to their associated protein product within the public Reactome pathway. Proteins in the pathway were color-coded according to how many DEGs contribute protein component(s) to each. Intracellular organelles or locations are labeled.

We next applied a robust signaling pathway enrichment algorithm to identify a total of 234 signaling pathways that were significantly affected at 24 hpi (Supplemental File 3). Seven significant pathways that were positively or negatively affected during the early stages of CHIKV infection in macrophages seemed especially relevant (Table 1). Collections of interacting proteins with the most significant corrected *p*-values also included Olfactory Transduction (Fig. 2), AGE-RAGE (Fig. 3), and Stimuli-Sensing Channels (Fig. 4). Our observation of the AGE-RAGE pathway is expected as it contains inflammatory proteins including interleukins, NF-κB, and JAK-STAT that regulate the acute inflammatory response and the innate immune system (Chen et al., 2018). The Olfactory Transduction pathway consists of many proteins that contribute to the regulation of Calcium signaling, presumably *via* Calmodulin. We also observed pathways involving Wnt signaling, extracellular matrix receptors, Insulin-like growth factor 1, Calcineurin, adherens junctions, and HIF-1-alpha (hypoxia) signaling.

**Table 1:** Top seven intracellular signaling pathways impacted by CHIKV in monocyte-derived human macrophages at 24 hpi.

Name of pathway	pSize*	NDE**	pGFWER^+^	Status^++^	Source database
**Stimuli-sensing channels**	100	76	2.71E−04	Activated	Reactome
**Wnt signaling pathway**	219	139	2.94E−04	Activated	Panther
**ECM-receptor interaction**	79	58	3.31E−04	Inhibited	KEGG
**IGF1 pathway**	30	22	0.000975	Activated	NCI
**Role of Calcineurin-dependent NFAT signaling in lymphocytes**	36	26	0.001006	Activated	NCI
**Stabilization and expansion of the E-cadherin adherens junction**	42	30	0.001025	Activated	NCI
**HIF-1-alpha transcription factor network**	64	44	0.001294	Activated	NCI

**Notes:**

* Number of genes assigned to the pathway.

** Quantity of DE genes found in each pathway.

+ Bonferroni-corrected *p*-value for each pathway.

++ The predicted direction of pathway modulation.

In an effort to identify DEGs that were shared across multiple pathways, we compared the members of multiple significant signaling pathways. We found that a subset of the modulated pathways shared DEGs including MAPK3 and MAPK1.

### Drug repurposing analysis identifies potential therapeutics for CHIKV

We cross-referenced all proteins in each significant signaling pathway against the opentargets.org database to identify known human drug targets within each significant signaling pathway and the associated existing small molecules that bind to each drug target in each pathway. We anticipated that this approach would identify drug targets that play a key role in fundamental viral processes, which would reduce virus replication and/or disease when targeted by a drug. As expected from the copious number of significant pathways, we predicted 136 pharmaceutical drugs from the 24 hpi pathways (Table 2). We then ranked these potential therapeutics with a novel toxicity ranking algorithm (Table 3). Briefly, this algorithm generates a single value that represents toxicity, safety, clinical trial status, and past repurposing efforts in virology. The top drugs predicted by our workflow included Telmisartan, Sunitinib, Etanercept, Vorinostat, Dasatinib, and Regorafenib as potential therapeutic drugs that could be repurposed to reduce signs, symptoms, and/or pathogenesis associated with infection by Chikungunya virus (Table 4).

**Table 2:** Therapeutics that significantly modulate the same pathways found in human macrophages infected with CHIKV at 24 hpi.

Drug name	ECM-receptor interaction	Influenza A	Salmonella infection	Olfactory transduction	Fc epsilon RI signaling pathway	Pathogenic *Escherichia coli* infection	Renal cell carcinoma	Chronic myeloid leukemia	Fc gamma R-mediated phagocytosis	AGE-RAGE signaling pathway in diabetic complications	Ras signaling pathway
**ALPELISIB**		X^4^			X^4^		X^4^	X^4^	X^4^	X^4^	X^4^
**IDELALISIB**		X^4^			X^4^		X^4^	X^4^	X^4^	X^4^	X^4^
**REGORAFENIB**		X^4^	X^4^		X^4^			X^4^		X^4^	X^4^
**MIDOSTAURIN**		X^4^				X^4^			X^4^	X^4^	X^4^
**DASATINIB**					X^4^			X^4^	X^4^		X^4^
**CANAKINUMAB**		X^4^	X^4^							X^4^	
**LUSPATERCEPT**							X^4^	X^4^		X^4^	
**AFLIBERCEPT**							X^4^			X^4^	X^4^
**RANIBIZUMAB**							X^4^			X^4^	X^4^
**BENZIODARONE**				X^4^							X^4^
**ETANERCEPT**		X^4^								X^4^	
**INFLIXIMAB**		X^4^								X^4^	
**COLLAGENASE CLOSTRIDIUM HISTOLYTICUM**	X^4^									X^4^	
**CONBERCEPT**										X^4^	X^4^
**TOFACITINIB**		X^4^								X^4^	
**IMATINIB**								X^4^			X^4^
**NETARSUDIL**			X^4^			X^4^					
**NILOTINIB**								X^4^			X^4^
**DIPYRIDAMOLE**				X^4^							
**ENTRECTINIB**											X^4^
**INTERFERON ALFA-2B**		X^4^									
**INTERFERON BETA-1A**		X^4^									
**INTERFERON BETA-1B**		X^4^									
**PANOBINOSTAT**								X^4^			
**PEGINTERFERON ALFA-2A**		X^4^									
**PEGINTERFERON ALFA-2B**		X^4^									
**PEGINTERFERON BETA-1A**		X^4^									
**PENTOXIFYLLINE**				X^4^							
**ROMIDEPSIN**								X^4^			
**RONTALIZUMAB**		X^4^									
**SIFALIMUMAB**		X^4^									
**SUNITINIB**											X^4^
**USTEKINUMAB**		X^4^									
**VORINOSTAT**								X^4^			
**ABEMACICLIB**								X			
**DOCETAXEL**						X^4^					
**PACLITAXEL**						X^4^					
**COLCHICINE**						X^4^					
**VEDOLIZUMAB**	X^4^										
**LEVETIRACETAM**	X^4^										
**OCRIPLASMIN**	X^4^										
**VINCRISTINE**						X^4^					
**HYDROXYCHLOROQUINE**		X^4^									
**NINTEDANIB**											X^4^
**ROXADUSTAT**							X^4^				
**AMISULPRIDE**											X^4^
**VALSARTAN**										X^4^	
**VINFLUNINE**						X^4^					
**ZOLEDRONIC ACID**		X^4^									
**LENVATINIB**											X^4^
**LITHIUM CARBONATE**		X^4^									
**NATALIZUMAB**	X^4^										
**SORAFENIB**											X^4^
**TIROFIBAN**	X^4^										
**VINORELBINE**						X^4^					
**ALENDRONIC ACID**		X^4^									
**DAPRODUSTAT**							X^4^				
**IMIQUIMOD**		X^4^									
**LOSARTAN**										X^4^	
**PAZOPANIB**											X^4^
**ANLOTINIB**											X^4^
**AXITINIB**											X^4^
**CD24FC**		X^4^									
**ERDAFITINIB**											X^4^
**ERIBULIN**						X^4^					
**INSULIN DETEMIR**											X^4^
**INSULIN GLARGINE**											X^4^
**IXABEPILONE**						X^4^					
**TELMISARTAN**										X^4^	
**VARESPLADIB METHYL**											X^4^
**VINBLASTINE**						X^4^					
**BARICITINIB**		X^4^									
**CABAZITAXEL**						X^4^					
**CABOZANTINIB**											X^4^
**ERLOTINIB**											X^4^
**GEFITINIB**											X^4^
**IBANDRONIC ACID**		X^4^									
**IBRUTINIB**					X^4^						
**INSULIN GLULISINE**											X^4^
**INSULIN LISPRO**											X^4^
**INSULIN PORK**											X^4^
**INSULIN SUSP ISOPHANE BEEF**											X^4^
**PONATINIB**								X^4^			
**RISEDRONIC ACID**		X^4^									
**TRASTUZUMAB EMTANSINE**						X^4^					
**VANDETANIB**											X^4^

**Table 3:** Predicted drug rankings by toxicity.

Drug name	Type	Clinically tested*	Withdrawn**	Virus application^+^	FDA approved	Chikungunya studies ^++^	Toxicity (Grayscale) ^+++^	Grade
**TELMISARTAN**	Small molecule	4		Yes	Yes	Yes	7	23.85714286^4^
**SUNITINIB**	Small molecule	4		Yes	Yes	Yes	7	23.85714286^4^
**ETANERCEPT**	Biotech: Protein base	4		Yes	Yes	Yes	6	23.83333333^4^
**VORINOSTAT**	Small molecule	3		Yes	Yes	Yes	6	23.83333333^4^
**DASATINIB**	Small molecule	4		Yes	Yes	Yes	5	23.8^4^
**REGORAFENIB**	Small molecule	4			Yes	Yes	2	18.5^4^
**CANAKINUMAB**	Biotech: Protein base	3		Yes	Yes		5	8.8^3^
**DIPYRIDAMOLE**	Small molecule	4		Yes	Yes		8	8.875^3^
**PENTOXIFYLLINE**	Small molecule	4		Yes	Yes		7	8.857142857^3^
**INFLIXIMAB**	Biotech: Protein base	4		Yes	Yes		7	8.857142857^3^
**ROMIDEPSIN**	Small molecule	3		Yes	Yes		3	8.666666667^3^
**LUSPATERCEPT**	Biotech: Protein base	3			Yes		8	3.875^2^
**ENTRECTINIB**	Small molecule	1			Yes		6	3.833333333^2^
**MIDOSTAURIN**	Small molecule	2			Yes		5	3.8^2^
**ALPELISIB**	Small molecule	2			Yes		4	3.75^2^
**PANOBINOSTAT**	Small molecule	3			Yes		2	3.5^2^
**IDELALISIB**	Small molecule	3			Yes		2	3.5^2^
**BENZIODARONE**	Small molecule	X	Yes				1	−9^1^

Grade Scale			
^1^unacceptable (<4)	^2^poor (5–20)	^3^acceptable (21–35)	^4^exceptional (36<)

**Notes:**

* Clinical trial phase for drug’s original indication.

** Drug was withdrawn from market.

+ Drug has been involved in previous antiviral studies.

++ Prior publications discussing the drug’s effectiveness against CHIKV.

+++ Toxicity of the drug determined by FD_50_ values and/or recommended diet restrictions, manufacturer safety statements, requirements of administration, and long-term impacts or risks that are included in drug consumption, all of these elements were evaluated by an assigned numerical value under the *Grayscale* (1–10 scale, where 1 is the most toxic).

**Table 4:** Top six ranked therapeutics for CHIKV.

Drug	Type	Clinically tested	Used with viruses	FDA approved	Chikungunya studies	Toxicity (Grayscale)	Grade
**TELMISARTAN**	Small molecule	4	Yes	Yes	Yes	7	23.85714286^4^
**SUNITINIB**	Small molecule	4	Yes	Yes	Yes	7	23.85714286^4^
**ETANERCEPT**	Biotech: Protein base	4	Yes	Yes	Yes	6	23.83333333^4^
**VORINOSTAT**	Small molecule	3	Yes	Yes	Yes	6	23.83333333^4^
**DASATINIB**	Small molecule	4	Yes	Yes	Yes	5	23.8^4^
**REGORAFENIB**	Small molecule	4		Yes	Yes	2	18.5^4^

## Discussion

The purpose of this study was to identify genes, functional annotations, and intracellular signaling pathways at 24 hpi that improve our mechanistic understanding of CHIKV infection of macrophages to aid in the prediction of prophylactics and/or treatments. To our knowledge, this is the first study to generate RNA-sequencing data from CHIKV-infected human macrophages. These cells play a key role in both the early stages of systemic spread in the human host, as well as the chronic sequelae after acute infection. Our results were also used to predict repurposed therapeutic drugs that could potentially be used as antivirals to combat CHIKV infection, replication, and/or pathogenesis in human macrophages. Throughout this study, we found that CHIKV-infected human macrophages modulate various biological pathways that generate intracellular and immune-related signals that likely contribute to the characteristic symptoms of fever, polyarthralgia, and/or chronic pain.

Interestingly, the lack of significant DEGs at eight hpi suggests at least two possible explanations: (1) a potential lag in the intracellular macrophage response to CHIKV infection, or (2) the selected MOI produced an infected macrophage population that was too small to confidently detect differential expression. Since CHIKV has a positive-sense genome, we do not expect this lagging response to be caused by a delay in viral genome replication. Rather, we believe that this observation is more likely due to the macrophages not initiating a response until after a sufficient number of CHIKV proteins have been produced in the cell. Prior studies have observed approximately 10^6^ plaque-forming units per mL and 10^6^ viral RNA copies per mL produced from cells infected at 0.1 MOI at 24 hpi in the same human monocyte-derived macrophage cell line (Guerrero-Arguero et al., 2020). Future experiments could provide additional insight into this hypothesis.

While this study focuses on the response of human macrophages infected with Chikungunya virus, prior CHIKV studies have independently validated a subset of our results. A mechanistic analysis of mouse samples identified various differentially expressed genes that are also present in our results, namely MFSD1, and FAM49B (Nakaya et al., 2012). Given that this prior study and ours were performed in different cell types, it is likely that these shared genes represent similar antiviral responses across multiple cell types.

Although minimal prior work has directly measured gene expression in CHIKV-infected macrophages, our observation of a larger number of DEGs being downregulated is somewhat expected given the primary immune functions performed by these host cells. Studies in other cell types have reported multiple cellular functions being inhibited by CHIKV infection including immune response (Selvamani, Mishra & Singh, 2014; Sharma, Balakathiresan & Maheshwari, 2015; Dhenni et al., 2021).

Prior studies examined the effect that CHIKV infection had on human skin fibroblast (HSF) cells, which showed several significant modulated signaling pathways at 24 hpi, particularly the “Wnt signaling”, “Stimuli-sensing channels”, and “ECM-receptor interaction” pathways (Komiya & Habas, 2008; Parashar et al., 2018; Roy, Byrareddy & Reid, 2020; Landers et al., 2021; Ghildiyal & Gabrani, 2021). These common pathways partially validate the relevance of our findings and may represent shared intracellular functions that contribute to the infected state of cells. The Olfactory Transduction pathway, which affects Calcium signaling, has been observed in prior work with Severe Acute Respiratory Syndrome Coronavirus 2 (SARS-CoV-2) and Vesicular Stomatitis Virus (Mishra, Byrareddy & Nayak, 2020; Krishnamoorthy et al., 2021). A previous study has shown that Calcium modulation can affect CHIKV replication in macrophages (Sanjai Kumar et al., 2021; Melton et al., 2002). The AGE-RAGE pathway, which contains JAK-STAT components, interleukins, and other proteins has not, to our knowledge, been directly reported in prior Chikungunya studies. Our findings are relevant given prior work showing the CHIKV nsP2 protein inhibits JAK-STAT signaling to regulate aspects of the inflammatory response (Fros et al., 2010), which contributes to many of the primary symptoms of CHIKV (Lu et al., 2015; Elgner et al., 2016).

Our subsequent analysis of DEGs and the associated signaling pathways identified potential therapeutic drugs that could be repurposed for CHIKV. Prior studies have reported progress on developing therapeutics that directly target viral proteins (Khan et al., 2010; Scuotto et al., 2017; Ho et al., 2018). In contrast, our drug repurposing analysis evaluated existing therapeutics that target human proteins that participate in key signaling pathways. A benefit of this approach is the reduced likelihood of the virus becoming resistant to such treatment since human genes mutate exponentially slower and are involved in key processes that play a role in virus replication.

Our approach to prioritizing drugs incorporated a novel toxicity metric to facilitate ranking of existing therapeutic drugs. Small molecules have been an area of active research for their anti-viral properties, and were purposefully targeted in our analysis due to their general chemical stability and minimal need for refrigeration during transport and/or storage. This is particularly important given that many CHIKV infections occur in geographical regions that may not have consistent access to cold-storage resources. Six of our drug predictions have already been found to reduce the effects of Chikungunya virus in cells including telmisartan, sunitinib, etanercept, vorinostat, and dasatinib (Bekerman et al., 2017; Dye, Brannan & Geneva Foundation Tacoma United States, 2017; Broeckel et al., 2019; Haese, Powers & Streblow, 2020) (Zaid et al., 2011; Roberts et al., 2015; Haridas & Haridas, 2019; “U18666A inhibits classical swine fever virus replication through interference with intracellular cholesterol trafficking,” 2019; Tripathi et al., 2020; De et al., 2021; Liang et al., 2019).

Vorinostat, trade name Zolina, is used to treat Cutaneous T-cell Lymphoma (CTCL) as a histone deacetylase inhibitor. Recently Vorinostat has performed well as an antiviral therapy for HIV and West Nile Virus (Lu et al., 2015; Wichit et al., 2017). Vorinostat enhances the antiviral qualities of U18666A, which is a multivesicular body (MVB) inhibitor that hinders the release of cholesterol from lysosomes. This effect has been observed to inhibit CHIKV replication in human skin fibroblasts (Lu et al., 2015; Elgner et al., 2016; Wichit et al., 2017; Ghildiyal & Gabrani, 2021).

Telmisartan, an angiotensin II receptor antagonist commonly known as Micardis or Pritor, was ranked the highest in our ranked results of repurposed drug candidates (PubChem, 2021). Recently, Telmisartan has risen in popularity from severe acute respiratory syndrome coronavirus-2 (SARS-CoV-2) clinical testing (Duarte et al., 2020; Rothlin et al., 2020). This drug has been shown to regulate glucose and lipid metabolism. Further testing is needed to determine whether any possible anti-inflammatory response effect is observed. A prior *in silico* drug repurposing study showed that Telistartan was predicted to have a docking affinity of −9.3 ± 0.1 kcal/mol to the CHIKV nsP2 protein (Montes-Grajales et al., 2020). Binding to a viral protein would be an added benefit as a potential therapeutic, particularly when combined with Novobiocin to inhibit nsP2 protease activity which causes the eventual dampening of the antiviral response (Montes-Grajales et al., 2020; Tripathi et al., 2020; Battisti, Urban & Langer, 2021).

Sunitinib, or Sutent, is a receptor tyrosine kinase inhibitor that affects protein translation, and is currently used to treat renal cancer (Bekerman et al., 2017). The testing of Sunitinib with Dengue virus showed reduced viral load in the serum and tissue (Pu et al., 2018). Sunitinib combined with Erlotinib can modify the inflammatory cytokine responses in models of dengue virus infection (Pu et al., 2018), which could also be relevant to CHIKV (Bekerman et al., 2017; Dye, Brannan & Geneva Foundation Tacoma United States, 2017; Pu et al., 2018). Sunitinib is currently being investigated in conjunction with oncolytic virotherapy (Kottke et al., 2010).

Dasatinib is a tyrosine kinase inhibitor that is commonly sold by the name Sprycel and is used for treating leukemia. This compound has shown promise as a prophylactic treatment for patients who are at-risk for HIV infection (Salgado et al., 2020). Results from the “Src Family Kinase Inhibitors Block Translation of Alphavirus Subgenomic mRNAs” demonstrated Dasatinib’s ability to directly affect viral RNA synthesis and decrease CHIKV RNA association with polysomes, indicating the importance of Src Family Kinase (SFK) activity in virus replication (Broeckel et al., 2019). Dasatinib has been found to block CHIKV replication and reduces infection-induced apoptosis in cells (Bekerman et al., 2017; Dye, Brannan & Geneva Foundation Tacoma United States, 2017; Haese, Powers & Streblow, 2020; Broeckel et al., 2019).

A multi-kinase inhibitor, Regorafenib (Stivarga), is used for the treatment of colorectal cancer and advanced gastrointestinal complications. It has a high level of toxicity and is only used after other treatments such as chemotherapy have been exhausted. This drug has anti-angiogenic properties and was shown to inhibit virus replication when administered prophylactically (Roberts et al., 2015). Sorafenib and Regorafenib can be used interchangeably, only differing in a single fluorine atom in their structures (Goel et al., 2018). A prior study confirmed that Sorafenib-treated cells suppress CHIKV replication (Roberts et al., 2015).

A variety of systemic clinical signs and symptoms are associated with CHIKV which are not limited to headache, myalgia, arthralgia, retro-orbital pain (Leo et al., 2009; Tan et al., 2018). Rash and other mucocutaneous signs, such as centrofacial hyperpigmentation, have also been observed and used as retrospective diagnosis (Mattar et al., 2015; Singal, 2017; Panigrahi, Chakraborty & Sil, 2021). Additional experiments will be needed to determine whether minimizing the infection and antiviral response in macrophages affects such systemic signs.

Past work has documented that patients infected with CHIKV can develop side effects or adverse reactions while undergoing long-term pharmaceutical treatments (Kim et al., 2016; Sil et al., 2021). We acknowledge that therapeutics can have unanticipated side effects when used *in vivo* and expect that subsequent validation experiments will be needed to show whether this is the case for the therapeutics we identified in this study.

Although this study primarily focused on predicting small molecule therapeutic treatments, an exception was made for Etanercept, a biotech fusion protein, because of its anti-inflammatory properties and acceptable safety profile (“Etanercept”; Jazwinski et al., 2011). Etanercept (Enbrel) manages a variety of inflammatory conditions including rheumatoid arthritis. Chikungunya virus has previously been characterized as having significant DEG overlap with rheumatoid arthritis (Nakaya et al., 2012; Suhrbier, 2019; Bautista-Vargas, Puerta-Sarmiento & Cañas, 2020). Although it is not recommended to treat patients who have pre-existing rheumatoid arthritis and are infected with the Chikungunya virus, otherwise-healthy patients could potentially benefit from receiving Etanercept treatments (Zaid et al., 2011; Haridas & Haridas, 2019). Future experiments will be required to determine the most effective dosage in human cells and to test the ability of these drugs to protect macrophages against CHIKV infection.

## Conclusions

Our analysis found zero significant DEGs at eight hpi in human monocyte-derived macrophages, but 9,676 genes, and eight GO terms in the macrophages at 24 hpi. We identified 234 biological pathways that were significantly altered in human monocyte-derived macrophages during CHIKV infection that represented AGE-RAGE, innate immunity, cell cycle-related pathways, and Calcium signaling. Our drug repurposing analysis predicted that a combination of Telmisartan, Sunitinib, and/or Dasatinib could be used as a potential therapeutic for CHIKV infection since together they modulate multiple intracellular host and viral processes in macrophages at 24 hpi. We expect that this study will contribute to ongoing efforts to develop effective prophylactic and/or therapeutic treatments for CHIKV.

## Supplemental Information

10.7717/peerj.13090/supp-1 Supplemental Information 1 List of differentially expressed genes from comparing 24 hpi *vs*. mock-infected cells.

10.7717/peerj.13090/supp-2 Supplemental Information 2 List of significant Gene Ontology terms from a comparison of 24 hpi *vs*. mock-infected cells.

10.7717/peerj.13090/supp-3 Supplemental Information 3 List of significant intracellular signaling pathways at 24 hpi.

## Additional Information and Declarations

### Data Availability

The following information was supplied regarding data availability: The differentially expressed genes, Gene Ontology enrichments, intracellular signaling pathways and the multiple hypothesis-corrected p-values are available in the Supplemental Files. The data is available at NCBI GEO: GSE182287.
